# Open questions on liquid–liquid phase separation

**DOI:** 10.1038/s42004-023-00823-7

**Published:** 2023-02-03

**Authors:** Evan Spruijt

**Affiliations:** Institute for Molecules and Materials, Radboud University Nijmegen, Nijmegen, the Netherlands

**Keywords:** Origin of life, Biophysical chemistry, Synthetic biology, Self-assembly

## Abstract

Liquid-liquid phase separation (LLPS) underlies the formation of intracellular membraneless compartments in biology and may have played a role in the formation of protocells that concentrate key chemicals during the origins of life. While LLPS of simple systems, such as oil and water, is well understood, many aspects of LLPS in complex, out-of-equilibrium molecular systems remain elusive. Here, the author discusses open questions and recent insights related to the formation, function and fate of such condensates both in cell biology and protocell research.

Liquid-liquid phase separation (LLPS) is such a common phenomenon in everyday life that we rarely think about it. Oil and water shaken together, for example when making a salad dressing, will demix in two coexisting liquid phases unless an emulsifier, such as pectin from mustard or lecithin from egg yolk, is added to stabilize the oil–water interface. Salt, sugars and spices added to the dressing distribute spontaneously between the phases depending on their molecular properties.

The same concepts—phase separation, interfacial tension, and partitioning—are not only important for good salad dressings, they are also involved in the organization and functioning of living cells, and are hypothesized to have played a role during the origins of life. Phase separation is believed to underlie the formation of various biomolecular condensates, membraneless inclusion bodies in the cell composed of mostly proteins and nucleic acids. Similarly, phase separation may have brought together molecular building blocks key to life inside a small compartment that resembled a cell: a protocell. However, there are differences with salad dressing: (proto)cells and their surroundings are chemically much more complex, containing numerous different molecules, surfaces, and out-of-equilibrium processes. Therefore, LLPS in the context of cellular organization and the origins of life still has many open questions with implications for chemistry, physics, and medicine.

From a chemistry point of view, intracellular phase separation differs from demixing of oil and water, because the proteins and nucleic acids involved in LLPS are water soluble and the condensates they form are strongly hydrated. This type of phase separation, in which solutes coagulate but remain in a liquid condensed state, was first described almost a hundred years ago, and termed coacervation. Interest in coacervation increased significantly more than a decade ago, when the same phenomenon was found to occur in cells. Recent studies have afforded a deeper understanding of coacervation, its driving forces and its role in biology, but also brought up new and intriguing questions, which are discussed here. We discuss questions on LLPS in relation to protocells and biomolecular condensates separately, as their chemistry differs significantly. Both sections are divided according to the stages of LLPS: the formation of phase-separated droplets, their function and composition, and their fate. Many aspects of LLPS, including LLPS-derived materials and extraction media, are beyond the scope of this work. This comment is concluded with a brief outlook of future applications for protocells and condensate research.

## Open questions on liquid–liquid phase separation for the origins of life

### Formation

**Fig. 1:**
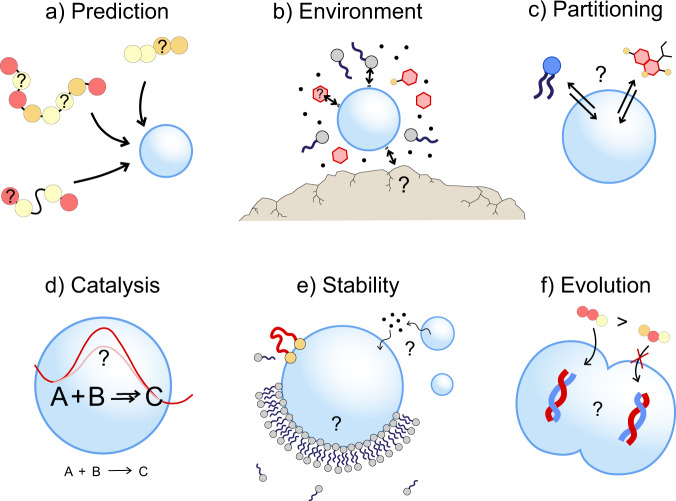
Open questions on phase-separated droplets and the origins of life. An increasing range of small molecules has been found to undergo LLPS to form coacervate protocells, but predicting a priori **a** the coacervation of molecules and **b** the role of environmental factors remains difficult. A molecular understanding of **c** partitioning and **d** catalysis inside coacervate droplets is lacking. Better understanding of **e** coacervate stability and the molecular structure of the interface would help to develop **f** increasingly complex, robust coacervate protocells that might ultimately proliferate and evolve.

Oparin first suggested that coacervation could have been a way to bring prebiotic molecules together and form a protocell. At that time only long polymers were known to form coacervates, either by complexation between oppositely charged polymers (complex coacervates) or by self-interactions and partial desolvation (simple coacervation). Since then, the scope of molecules capable of LLPS has been broadened significantly, and the molecular rules for phase separation have been largely established. While this is particularly true for complex coacervates, the prediction of simple coacervation remains elusive and there are only few examples of simple coacervates from short peptides and other small molecules^1–3^. A recent study proposed a promising strategy to distinguish liquid from solid condensates in molecular dynamics simulations of dipeptides based on exchange dynamics, although the precise boundaries between soluble peptides, coacervates, and aggregates were fuzzy^4^. Accurately predicting LLPS and defining molecular rules for simple coacervation of larger peptides and other molecules remains an open challenge (Fig. 1a).

By extension, the influence of other solutes on LLPS is often far from clear. Mono- and divalent salts are commonly known to destabilize complex coacervates, but glutamate and aromatic sulfonates surprisingly enhance LLPS of proteins. Moreover, mixtures of phase-separating molecules can exhibit synergy or interference of LLPS. In general, multicomponent mixtures are more likely to separate in multiple coexisting phases and those phases are more robust to fluctuations in the number or concentration of components^5^. Nevertheless, a deeper understanding of LLPS and multiphase organization in complex mixtures is required, as protocell organization may underlie their function as reactors.

Finally, the presence of surfaces, gradients, and energy sources can all influence LLPS in unexpected and nontrivial ways. Surfaces, for example, can promote LLPS through heterogeneous nucleation, but also suppress LLPS by decreasing the saturation concentration in solution via adsorption (Fig. 1b). Gradients and energy dissipation can bring LLPS systems out of equilibrium: an exciting area, in which new phenomena can emerge, such as self-selection and growth and division, that require further investigation^6–8^.

### Function and composition

One reason why Oparin proposed coacervates as promising protocells is their ability to concentrate molecules key to life and act as catalysts of reactions between those molecules. Most solutes can enter coacervates without barrier and their distribution is ideally governed by partitioning. Because coacervates typically contain a significant fraction of organic matter, they can favorably interact with a wide range of organic solutes, resulting in elevated internal concentrations. However, beyond some basic rules of thumb^9^, there is limited understanding of the quantitative laws of partitioning into coacervates (Fig. 1c). Experimentally, systematic partitioning studies are lacking, and similar probes measured in different coacervates sometimes show wide variations in partitioning or adsorption at coacervate interface^1^. Theoretically, a quantitative description of partitioning is also challenging. Partitioning equilibria typically assume thermodynamically ideal behavior in both phases and ignore saturation effects and the coacervate’s internal microstructure.

Locally increased concentrations as a result of partitioning can lead to higher rates of chemical reactions inside coacervates. In addition, the altered chemical microenvironment inside the coacervate can also change the relative energy levels of substrate, product and transition states, thereby changing the effective rate constants of chemical reactions (Fig. 1d)^9^. This makes coacervates effective catalysts, and uncovering their catalytic function in different types of chemical reactions is an important open question. Recently, a simple coacervate was reported to enhance an aldol condensation reaction of two small molecules, via a combination of concentration and catalysis^1^. In complex coacervates, a reduced charge density of the phase-separating peptides was found to result in higher ribozyme activity^10^. Finally, accumulation of a labile imine in H-bond coacervates beyond the increased equilibrium constant suggested that coacervates could also facilitate multistep reactions by stabilizing kinetically labile intermediates^11^. These examples illustrate that coacervates can profoundly alter chemical reaction kinetics, in particular of larger reaction networks. A more detailed chemical understanding of these effects require further experiments.

### Fate

One of the most interesting and challenging questions about the role of LLPS-based protocells in the origins of life is how protocells could have gained increasing complexity and life-like functionalities^12^. A common problem of membraneless protocells is their poor stability—against ripening and coalescence, spreading on surfaces and dissolution. How could coacervates have survived long enough to play a role in the emergence of life? Recently, it was reported that complex coacervates show no signs of Ostwald ripening, possibly because of the complexed nature of their contents^6^. Peptides, proteins, lipids, and even small molecules can also adsorb at the surface of some coacervates and effectively stabilize them^13,14^. And spreading of coacervates at an air-water interface could lead to droplet splitting, thereby providing a way to proliferation^8^. These are remarkable findings that urge for a better understanding of coacervate stability and the molecular structure of the interface (Fig. 1e)^15^.

Finally, if coacervate protocells remain more stable than commonly thought, it is interesting to consider mechanisms that lead to the evolution of increasingly life-like functionalities. Can coacervate protocells selectively multiply a subset of molecules? Can coacervates move directionally, triggered by chemical signals, in a chemotactic fashion? Can molecular information be stored and replicated in coacervates (Fig. 1f)? These questions give some outlines of a broad, largely unexplored field of endowing LLPS-based protocells with additional hallmarks of life, in which different branches of chemistry come together.

## Open questions on liquid–liquid phase separation in cell biology

### Formation

Liquid–liquid phase separation of proteins and RNA underlies the formation of intracellular membraneless compartments, also known as biomolecular condensates. Because phase-separating proteins are typically much larger than the small molecules used for coacervate protocells, classical models from polymer physics have been used to qualitatively describe LLPS of proteins. Prediction of phase-separating sequences has also significantly improved in recent years, on account of machine learning methods^16^. However, a closer inspection also reveals important aspects of condensate formation that are not yet taken into account.

**Fig. 2:**
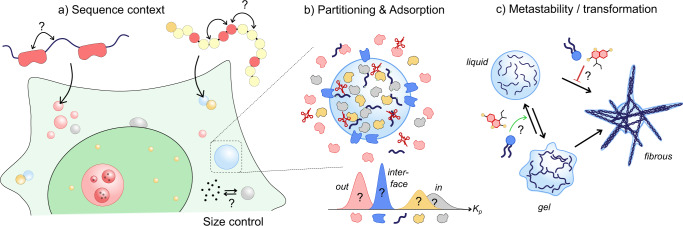
Open questions on phase-separated droplets in cellular organization. Predicting phase-separating protein sequences is increasingly successful, **a** the chemical context provided by surrounding amino acids, crowding, and active energy-driven processes are not well understood yet. **b** The precise composition of condensates and their interfacial structure is often not known, but may have important implications for their function. **c** Condensates can undergo further transformation into gel-like structures or fibrous aggregates, but the underlying energy landscape and strategies to modulate it remain to be uncovered.

Molecular predictors place emphasis on identifying so-called stickers or interaction motifs in protein sequences as drivers of LLPS. However, recent work showed that the interaction strength of sequence-specific motifs is dependent on the chemical context provided by the surrounding amino acids and that sequence and chemical context can be compensatory and synergistic (Fig. 2a)^17^. The theoretical models that describe the phase behavior of a given protein sequence are also limited: they describe equilibrium phase separation of single components, while the cellular context is out-of-equilibrium and crowded. To what extent active processes^18^ and crowding^19^ determine biocondensate formation, organization and properties remain unclear. Studies of temperature-dependent condensate formation in vivo suggest that both active processes and passive thermodynamics govern the assembly of nucleoli^20^, but similar studies on other proteins and condensates are required to answer this question in general.

Finally, it has been noted that the evidence for LLPS occurring in vivo is often phenomenological and based on observations of subdiffractional spots^21^. This point is connected to another open question about the size regulation of biomolecular condensates. Condensates nucleate as small clusters of proteins that may grow into larger condensates or remain as small clusters by active modifications or chaperone activity^22^. Understanding the fundamental and functional differences between protein assemblies and (small) phase-separated condensates, and how cells control the size of the latter is important for a better understanding of the role of biomolecular condensates in cellular chemistry.

### Function and composition

Partitioning, combined with interfacial adsorption, governs the distribution of client molecules in biomolecular condensates, like for coacervate protocells. Determining their composition often holds the key to understanding their function. However, quantifying the distribution of clients is even more difficult for condensates in cells than for protocells in a test tube, as they are small and easily disrupted. Therefore, the precise composition of many condensates is not known (Fig. 2b). Recently, proximity labeling using biotin phenol radicals has been used to label condensate components in situ, enabling enrichment after cell lysis and detection by mass spectrometry^23^. This method holds great promise to identify key condensate components in healthy conditions and in LLPS-related diseases.

Proteins can also adsorb to the condensate interface, as was recently observed for coacervates^14^, and is common for oil droplets in water. Given the large number and variety of proteins in the cell, it seems inevitable that many condensates have a characteristic peripheral layer containing certain adsorbed proteins. Such a layer can have important implications: it stabilizes condensates, but it could also alter their ability to sequester clients and destabilize the adsorbed proteins. Interestingly, the adsorption of protein clusters to condensate interfaces was recently discovered to control condensate dynamics and slow down coarsening in cells^24^. Finally, condensate adhesion to membranes can also affect their dynamics and function. However, we are only beginning to discover the functional consequences of interactions between condensates and surfaces such as membranes and protein filaments^25,26^.

### Fate

Condensates can undergo further (irreversible) transitions into gel-like states or fibrillar aggregates, a process that is called aging^27^. Such transitions can originate from phase-separating, prion-like proteins in the condensates that undergo a liquid-to-solid transition (LST), or from amyloidogenic proteins that partition into the condensates and aggregate. LST has been implicated in various diseases, making a fundamental understanding pertinent. However, many simpler coacervate models do not exhibit equivalent LST, which raises the question of what the minimal requirements are for condensate LST. Aging involves protein conformational changes and diffusion in a dense, crowded environment and following a highly complex energy landscape. Moreover, LST can have different forms and result in gel-like structures, amorphous solids, structured fibers, or something in between (Fig. 2c). Model systems that can undergo a form of LST would help to map the contours of the energy landscape and unravel the mechanism behind LST. Finally, such model systems can also help chemists to design and screen compounds that can modulate condensate aging^28^, which has important prospects for therapeutics.

## Outlook

Progress in the synthesis of biomolecules, modeling, and microscopy have greatly improved our understanding of phase separation in complex, aqueous media, such as the interior of a cell. The coming years will surely see more of the complexity being mastered and LLPS-based compartments utilized in innovative applications, such as direct delivery into (synthetic) cells^2^ or catalysts for green chemistry^1^.
